# Ultrastructural membrane dynamics of mouse and human cortical synapses

**DOI:** 10.1101/2024.12.26.630393

**Published:** 2024-12-26

**Authors:** Chelsy R. Eddings, Minghua Fan, Yuuta Imoto, Kie Itoh, Xiomara McDonald, Jens Eilers, William S. Anderson, Paul F. Worley, Kristina Lippmann, David W. Nauen, Shigeki Watanabe

**Affiliations:** 1 Department of Cell Biology, The Johns Hopkins University, Baltimore MD, 21205, USA.; 2 Solomon H. Snyder Department of Neuroscience, The Johns Hopkins University, Baltimore MD, 21205, USA.; 3 Carl-Ludwig-Institute of Physiology, Faculty of Medicine, Leipzig University, Leipzig 04103, Germany.; 4 Department of Neurosurgery, The Johns Hopkins Hospital, Baltimore, MD, 21205, USA.; 5 Department of Neurology, The Johns Hopkins Hospital, Baltimore, Maryland, 21205, USA.; 6 Department of Pathology, The Johns Hopkins Hospital, Baltimore, Maryland, 21205, USA.; 7 The Center for Cell Dynamics, The Johns Hopkins University, Baltimore, MD, 21205, USA.

**Keywords:** synaptic transmission, time-resolved electron microscopy, zap-and-freeze, cortex, high-pressure freezing, synaptic vesicle endocytosis, ultrafast endocytosis, human neocortex, stimulated emission depletion microscopy, Dynamin 1xA, cerebellum, 2-photon calcium imaging

## Abstract

Live human brain tissues provide unique opportunities for understanding the physiology and pathophysiology of synaptic transmission. Investigations have been limited to anatomy, electrophysiology, and protein localization—while crucial parameters such as synaptic vesicle dynamics were not visualized. Here we utilize zap-and-freeze time-resolved electron microscopy to overcome this hurdle. First we validate the approach with acute mouse brain slices to demonstrate that axons parallel to the electrical field can be stimulated to produce calcium signaling. Next we show that ultrafast endocytosis is induced and can be captured in both mouse and human brain slices. Crucially, in both species a protein essential for ultrafast endocytosis Dynamin 1xA (Dyn1xA) localizes to the region peripheral to the active zone, the putative endocytic zone, indicating a likely mechanism conservation between mouse and human. This approach has the potential to reveal dynamic, high-resolution information about synaptic membrane trafficking in intact human brain slices.

## Introduction

Direct investigation of synaptic transmission in human brain tissues will increase our understanding of typical brain states and how they are impacted by age and disease. Dynamic measures of membrane properties and firing patterns of human neurons are observed via electrophysiology of *ex vivo* brain slices. Electrophysiology enables quantitative evaluation of synaptic properties of specific neuronal subtypes in particular brain regions. For example, human neurons located in different cortical layers exhibit heterogeneous morphologies, passive membrane and suprathreshold properties, as well as firing rates—with layer 5 pyramidal neurons presenting higher frequency firing rates compared to layer 2/3 and layer 3c^1^. These properties can also change across different time scales. For instance, the resting membrane potential and resistance of layer 2/3 pyramidal neurons differ from infancy to old age^2^. Consequently, electrophysiology provides information on quantal content, evoked and spontaneous vesicular release, and the plastic nature of synaptic transmission. Likewise, this method reveals species specific differences in synaptic reliability and circuit connectivity between mice and humans. Human cortical pyramidal synapses are more reliable and less likely to fail compared to mouse pyramidal synapses—with a failure rate of 0% compared to 25% in mice^3^. Work on human CA3 hippocampal tissues suggest higher temporal precision in human synapses compared to rodent synapses. Human hippocampal synapses show pronounced adaptation, more narrow interspike intervals and overall lower synaptic fluctuations^4^. Yet, relying solely on electrophysiology cannot easily discern the anatomical and structural features of synapses.

Cellular and synaptic morphologies are often characterized by electron microscopy (EM). Enhanced resolution clearly distinguishes presynaptic from postsynaptic compartments and reveals the relative locations of organelles. An advantage of EM is that it yields non-targeted information about the morphology of all cells in a tissue while preserving their spatial relationships^5^. Moreover, electron micrographs provide detailed information about how neurological diseases impact non-neuronal cells and myelination in patient brains. For example, post-mortem Alzheimer’s patient hippocampal tissues contain ‘dark astrocytes’ showing signs of cellular stress like altered mitochondria and electron-dense plaques^6^. On the other hand, examinations of post-mortem Multiple Sclerosis patient brains show reduced myelination and possible compensatory axonal swelling in regions of normal appearing white matter^7^. Along with the advent of volumetric imaging and machine-learning based segmentation approaches, millions of human synaptic connections are now being mapped. Recently a petascale dataset was published from a 1 mm^3^ human cortical biopsy illustrating vasculature, rare multisynaptic connections, and intact surrounding tissue^8^. Other EM datasets reveal subtle connections between human neurons and glia through cilia-based contacts^9^, and a 10-fold expansion of the interneuron-to-interneuron network in humans compared to mice^10^. Overall, ultrastructural investigations of human brain tissues provide additional insights into how synaptic circuits are situated within the larger cerebral context. However, electron micrographs are static images of tissue and do not provide information on synaptic release properties beyond the distribution of organelles.

These various methods of studying synapses can require vastly different sample preparation requirements, resulting in a frequent ‘structure-function’ gap in understanding synaptic transmission. Time-resolved electron microscopy is situated as a potential resolution to this structure-function dilemma. Combining optogenetic stimulation and high-pressure freezing ‘flash-and-freeze’ EM has been successfully used in: *C. elegans* neuromuscular junctions^11^, cultured mouse hippocampal neurons^12^, acute mouse brain slices^13^, and mouse hippocampal organotypic slice cultures^14^. An advantage and restriction of flash-and-freeze is its reliance on channelrhodopsin activation of neurons. Channelrhodopsin precisely activates select circuits through restricted expression to a subset of neurons^13,14^. This exogenous system can limit users to transgenic mouse lines or virally infected tissues. In addition, the number of elicited action potentials in each neuron may be variable due to differential expression levels^12^. Although human organotypic slice cultures can be transduced with channelrhodopsin expression^15^, long-term culture likely requires readily accessible human cerebrospinal fluid sources^16^ and could alter synaptic properties. Therefore, an approach suitable for acutely resected tissue is necessary.

Here, we adapt ‘zap-and-freeze’ EM for use with acute brain slices as another modality to bridge form and function, yielding functional and ultrastructural information about presynaptic membrane trafficking. Zap-and-freeze EM uses transient electric field stimulation to activate neurons before high-pressure freezing^17^. By controlling the stimulation paradigm as well as the time interval between stimulation and freeze, snapshots of dynamic events are captured with millisecond temporal resolution. We applied zap-and-freeze EM to acute mouse brain slices and acute human neocortical slices obtained from epilepsy surgeries. We demonstrate that synaptic activities can be induced in slices loaded on the zap board for a high-pressure freezer. Our results indicate that synaptic vesicle recycling in human neocortical presynapses is likely mediated by ultrafast endocytosis, owing to a depot of the protein Dynamin 1xA (Dyn1xA), similar to mouse hippocampal synapses. Our approach can enable ultrastructural characterizations of activity-dependent membrane trafficking at synapses in acute brain slices.

## Results

### The ‘zap board’ induces calcium signaling in acute mouse brain slices

**Figure 1.**
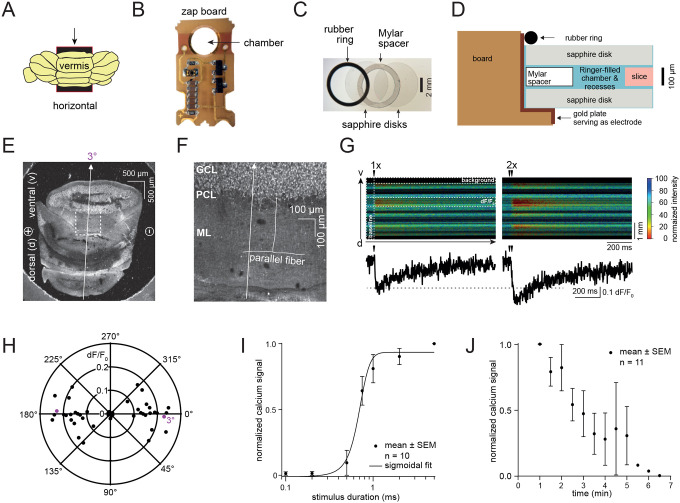
2-photon calcium imaging of acute mouse cerebellar slices on the zap board. (A) Acute mouse cerebellar slices were prepared by sectioning the cerebellar vermis horizontally to produce axons with known orientation. Arrow indicates vibratome cut direction. (B) A zap board enabling electrical stimulation in the Leica EM ICE high-pressure freezer. (C) Overlay image of a 6 mm wide rubber O-ring, two sapphire disks and a Mylar spacer ring in between two sapphire disks. When assembled, a specimen is sandwiched between two sapphire disks. (D) A schematic showing another view of the assembly holding the slice in the zap board chamber. (E) Horizontal 100 μm cerebellar slice lying between two sapphire discs and a Mylar spacer ring for 2-photon imaging on the zap board. Parallel fibers (PF) from the ascending axons of granule cells (GCs) are in line with the electrical dipoles ((−) to (+)) and were imaged orthogonally in the line-scan mode (arrow, 3° deviated in this example). (F) Zoom-in image of a slice indicating the PFs in the molecular layer (ML), arising from GCs in the granule cell layer (GCL). An arrow depicts the orientation of the line scan. PCL: Purkinje cell layer. (G) Normalized Fura-2, AM signal after a single (1x) and two consecutive (2x, 50 ms apart) stimuli. (H) Polar coordinate plot of normalized Ca^2+^ signal vs. linescan / PF orientation. 0/180° account for PFs lying in line with the electrical dipole, while at 90/270° PFs lie orthogonally to the dipole. Circles indicate Ca^2+^ signal strength. n=25 slices from 4 animals. (I) Plot showing the normalized Ca^2+^ signal versus stimulus duration. Data are presented as mean ± SEM. n=10 slices from 2 animals. (J) Plot showing the normalized Ca^2+^ signal versus time after assembling the slice in the zap board chamber. Data are presented as mean ± SEM. n=11 slices from 2 animals.

Towards our goal of visualizing synaptic vesicle dynamics in human tissues, we began by determining if the microcircuit chip used in zap-and-freeze (called the ‘zap board’ hereafter) is compatible with brain slices. The board’s circuit diagram and detailed electrical properties are described in Figure S1A–E. Originally, the zap board was constructed to apply a 10 V/cm electric field to induce action potential-driven synaptic activities in cultured mouse neurons^18^. Provided the orientation of axons is highly variable in culture, most neurons within the electric field can be stimulated^18^. The orientation of axons in an intact neural circuit may influence the generation of action potentials in slices^19,20^. Therefore, we tested the zap board using axons with a well-defined circuit orientation in slices: the parallel fibers of granule cells, which form synapses with Purkinje cells, in the mouse cerebellum (Figure 1A).

To visualize neuronal activity, we performed 2-photon calcium imaging in acute mouse cerebellar slices. Slices were placed between two sapphire disks, separated by a 100 μm Mylar spacer ring within the zap board (Figure 1B–D). The zap board is normally activated by pulses of blue light of various durations^18^ (typically 1 ms is sufficient to induce an action potential in cultured neurons^21^). To avoid blue light interference during 2-photon imaging when activating the zap board, we relocated the photodiode to an external optocoupler and drove the board with transistor-transistor logic (TTL) signals from a patch-clamp amplifier. In this setup, the zap board remains optically activated with blue light, as it is in the high-pressure freezer, but can now be placed on a 2-photon laser-scanning microscope (Figure S1). After determining the optimal stimulus parameters (e.g., 2.55 V LED command voltage) in this system with external resistances (Figure S1), we tested the electrical stimulation on slices.

For calcium imaging, 100 μm mouse cerebellar slices were incubated with the membrane-permeable calcium indicator Fura-2, AM (which darkens with increasing calcium concentrations) and mounted on the zap board (Figure 1B–D). First, we oriented cerebellar slices such that the parallel fibers were themselves parallel with the electric field (3°, Figure 1E,F). Following a single command voltage applied to the LED to activate the zap board for 1 ms, calcium transients were successfully detected in the molecular layer (ML; Figure 1G). Calcium signaling was enhanced when two consecutive 1 ms pulses were applied 50 ms apart (Figure 1G), indicating synaptic activity in parallel fibers^22^ is successfully induced by the zap board. To test the effect of orientation within the zap board, we varied the angular orientation of brain slices relative to the device’s electric dipoles. Calcium imaging suggested that neuronal signaling was greatest when the parallel fibers were in line with the electric dipole (0°) and decreased progressively as the angle changed to 45° (Figure 1H). Beyond 45°, essentially no calcium signal was detected (dF/F_0_ close to 0, Figure 1H), suggesting that the parallel fibers were not sufficiently stimulated. Interestingly at 90°/270° where ascending axons should be in line with the electric field, no calcium signal was observed—likely because superficial parallel fibers were imaged, whose ascending axons were cut during the slicing procedure. Nonetheless, these data suggest brain slices can be activated on the zap board with a single 1 ms pulse.

Next, we varied the electric pulse duration and the incubation time of the slice in the chamber of the zap board, while keeping the slice at 0° relative to the dipoles. When an electric pulse was applied for 0.1–0.5 ms, which is typically used in electrophysiology experiments^23–25^, almost no signal was detected (Figure 1I). The calcium signal nearly peaked with a 1 ms pulse (Figure 1I), suggesting that a 1 ms electrical pulse is sufficient for action potential induction or, at least, the detection of calcium signals in this setup. Finally, to address how long slice health can be maintained on the zap board, we measured the calcium signals every 30 s over 7 min after a top sapphire disk was placed over the brain slice (Figure 1C,D, and J). For the first 2 min, calcium signaling was maintained. However, beyond 2 min, signals were drastically decreased, and by 6 min undetectable (Figure 1J). Thus, for subsequent ultrastructural experiments we applied a 1 ms electric field pulse to the specimen and discarded all specimens not frozen within 2 min after they were enclosed within two sapphire disks.

### Visualizing synaptic vesicle endocytosis in acute mouse brain slices

As human brain samples are rare and precious, we initially explored membrane dynamics using zap-and-freeze in acute mouse brain slices. Instead of cerebellar regions, we prepared cortical slices as many available live human brain tissues are neocortical. Following slicing and a slightly modified NMDG recovery^26^ (see Methods), we mounted each brain slice such that the cortical layers were parallel to the zap board’s electric field. A single 1 ms stimulus was given, and individual slices frozen at 100 ms, 1 s and 10 s. The selected time points were previously used to visualize synaptic vesicle recycling in cultured hippocampal neurons^12,27–31^ and can potentially capture several endocytic pathways (including kiss-and-run, ultrafast endocytosis, and clathrin-mediated endocytosis)^12,32^. Fluid phase makers of endocytosis such as cationized ferritin^12,31^ were not used on brain slices since their tissue penetration would not be sufficiently uniform.

**Figure 2.**
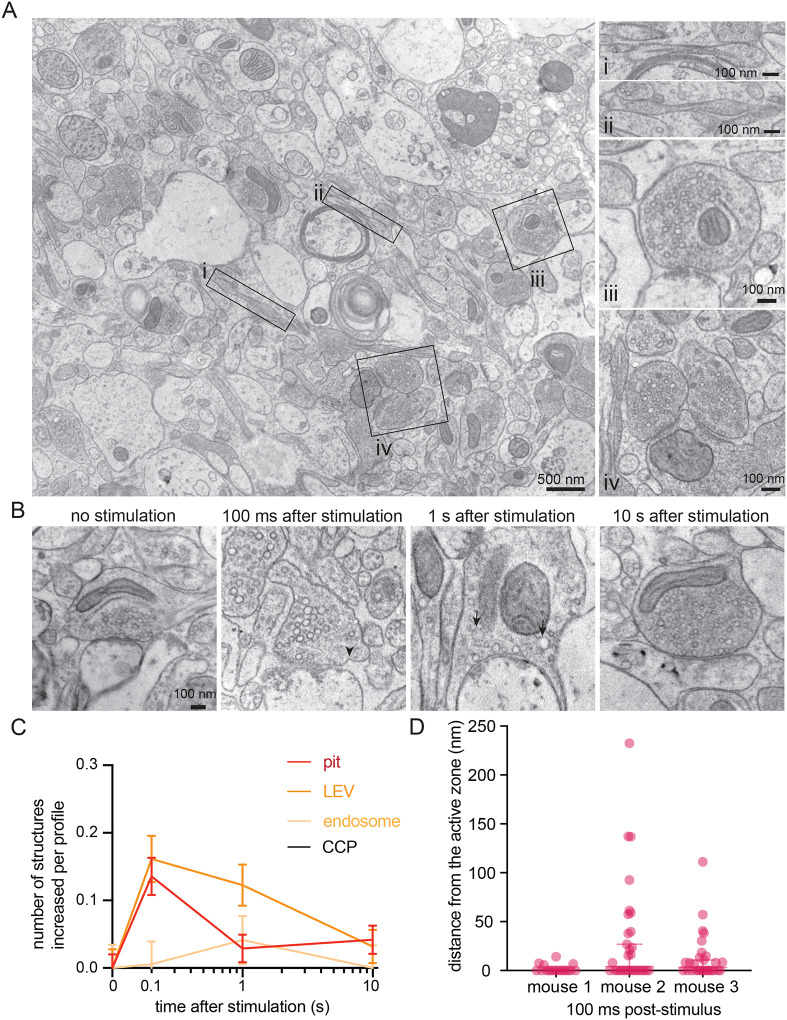
Ultrafast endocytosis in cortical synapses of acute mouse brain slices. (A) Low magnification overview of an acute mouse brain slice visualized with zap-and-freeze EM (from an unstimulated slice). Axons with pearled morphologies are highlighted as panels (i and ii), along with examples of synapses (iii and iv). (B) Example electron micrographs showing endocytic pits (black arrowheads) and putative large endocytic vesicles (LEV) and endosomes (black arrows) at the indicated time points in cortical regions of acute mouse brain slices. More example TEM images are provided in Figure S2. (C) Plots showing the increase in number of each endocytic structure per synaptic profile after a single stimulus. Data are pooled from three experiments and presented as mean ± SEM. CCP: clathrin-coated pits. See Data Table S1 for n values and detailed numbers for each time point. (D) Plot showing the distance distribution of putative endocytic pits from the edge of an active zone 100 ms post-stimulus in acute slices from n=3 mice. Data are presented as median ± 95% confidence interval. Each dot represents a pit.

Tissue gross morphologies were left largely intact (Figure 2A) when passed through the high-pressure freezer using 15% polyvinylpyrrolidone (PVP) as a cryoprotectant—chosen because of its past success in flash-and-freeze^13^. Similar to dissociated hippocampal neurons and slices, mouse cortical axons exhibited a pearled morphology^33^ within acute slices (Figure 2A i and ii). Ultrastructural analysis of synapses revealed uncoated pits forming 100 ms post-stimulus (Figure 2B,C, Figure S2A–C for additional images). These pits appeared at putative ultrafast endocytosis sites—primarily within a range of 0–4 nm from the edge of an active zone, defined as the membrane region opposite the postsynaptic density^12,31^ (Figure 2D; median pit distances: 0, 3.892, and 3.142 nm for mouse 1, 2, and 3 respectively). The number of large vesicles (diameter over 60 nm) also peaked at 100 ms, followed by a slight increase in the number of endosomes (spherical organelles with diameter 100 nm or more, or oblong organelles larger than synaptic vesicles) 1 s post-stimulus (Figure 2B,C; Figure S2A–C for additional micrographs). No apparent clathrin-coated pits or Ω-structures indicative of potential kiss-and-run like events^34–36^ formed at synapses at any of the tested time points (Figure 2C; see Figure S2D for micrographs of uncoated pits versus one observed clathrin-coated pit in a dendrite from this dataset for comparison). These results suggest that ultrafast endocytosis is likely a major endocytic pathway in cortical synapses after a single stimulus.

**Figure 3.**
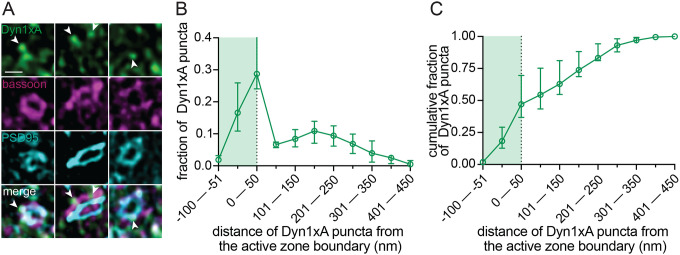
Dyn1xA clusters next to an active zone in mouse cortical synapses. (A) Example STED images of endogenous Dyn1xA localizations (white arrowheads) in cortical regions of acute mouse brain slices. Scale bar: 300 nm. Dyn1xA antibody confirmation in Figure S3. More example STED images provided in Figure S4. (B) The distribution of Dyn1xA puncta relative to the active zone edge, defined by Bassoon, analyzed in top view synapse images. Shaded region indicates area inside the active zone. The median and 95% confidence interval are shown for n=3 mice; see Data Table S1 for specific n values. (C) Cumulative plots of data presented in (B).

To verify that the observed uncoated pits possibly represented ultrafast endocytosis intermediates, we localized a protein crucial for ultrafast endocytosis, Dyn1xA, in acute brain slices using stimulated emission depletion (STED) microscopy. The kinetics of ultrafast endocytosis are produced by pre-recruitment of endocytic proteins at the endocytic zone^29,30^. Dyn1xA molecules are therefore expected to localize next to the active zone of synapses at rest if those synapses can perform ultrafast endocytosis. Previous studies of Dyn1xA mainly relied on tagged protein constructs that were either overexpressed or genetically knocked-in to neurons^30,37^. To examine endogenous protein localizations in slices, we developed and knock-out validated a rabbit polyclonal antibody against a Dyn1xA specific C-terminal 20 amino acid region, which interacts with Endophilin A1 for ultrafast endocytosis^29^ (Figure S3A–D). Immunoblotting showed that the Dyn1xA antibody reacted with lysate from mouse neurons but not astrocytes (Figure S3A). The selected amino acid region is 100% identical between mouse and human (Figure S3D), and this antibody recognized Dyn1xA protein in mouse and human whole brain lysates (Figure S3C). Analysis of mouse cortical synapses showed punctate Dyn1xA antibody signals present in PSD95-positive excitatory synapses (Figure 3A; Figure S4 for example mouse slice STED overview and additional side view synapses that were present but not analyzed). Similar to cultured hippocampal neurons, Dyn1xA puncta were found directly at the active zone boundary and periactive zone (−50 nm to +50 nm) (Figure 3B), with nearly 47% of Dyn1xA signals localized within this region in excitatory cortical synapses (Figure 3C). These results suggest that Dyn1xA is present at the endocytic zone and that ultrafast endocytosis likely mediates synaptic vesicle recycling in rodent cortical synapses within an intact slice.

### Ultrafast endocytosis in human cortical synapses

Having verified the zap-and-freeze approach in mouse cortical slices, we assessed its utility in human brain tissue. We received tissues from four epilepsy patients undergoing surgery (see Table S3 for de-identified patient information). These tissues originated from non-affected cortical areas removed to access hippocampus—this tissue removal was a required action in the overall surgery. We prepared 100 μm slices and performed our slightly modified NMDG recovery (see Methods for details, Figure S5A). To assess the viability and health of the slices, we performed electrophysiology for two cases. Recovered human pyramidal neurons (from 1 female and 1 male) showed typical action potential traces in response to depolarization, ability to respond to current steps (Figure S5B–G), and solid membrane properties with slight inter-individual variability as expected (Table S2). The recorded neurons also exhibited normal morphology, assessed with post-recording biocytin cell-fill staining (Figure S5H), indicating the tissues were viable and healthy.

**Figure 4.**
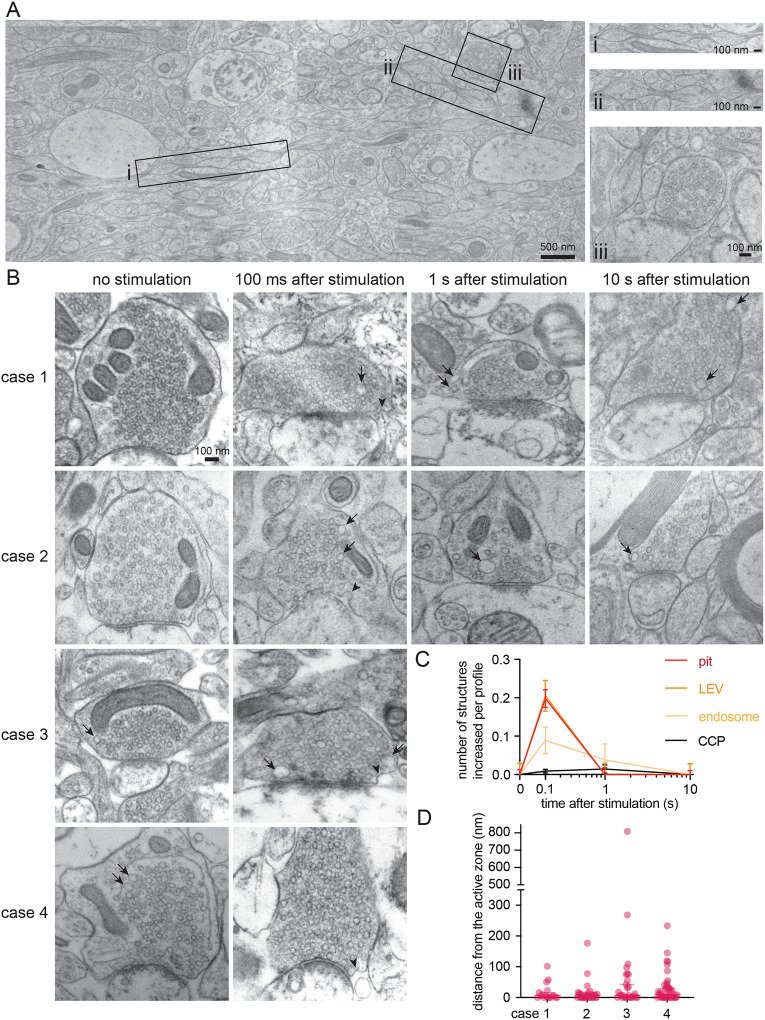
Ultrafast endocytosis in cortical synapses of acute human brain slices. (A) Low magnification overview of acute human neocortical slice visualized with zap-and-freeze EM (from a slice frozen 10 sec post-electric field stimulation). Axons with pearled morphologies are highlighted as panels (i and ii), along with an example of a synapse (iii). (B) Example electron micrographs showing endocytic pits (black arrowheads) and putative large endocytic vesicles (LEV) and endosomes (black arrows) at the indicated time points in acute brain slices from four humans. Electrophysiological confirmation of human brain slice electrical viability in Figure S5. More example TEM images provided in Figure S6. (C) Plots showing the increase in number of each endocytic structure per synaptic profile after a single stimulus. Data are presented as mean ± SEM. CCP: clathrin-coated pits. See Data Table S1 for n values and detailed numbers for each time point. (D) Plot showing the distance distribution of putative endocytic pits from the edge of an active zone 100 ms post-stimulus in acute neocortical slices from n=4 humans. Data are presented as median ± 95% confidence interval. Each dot represents a pit.

**Figure 5.**
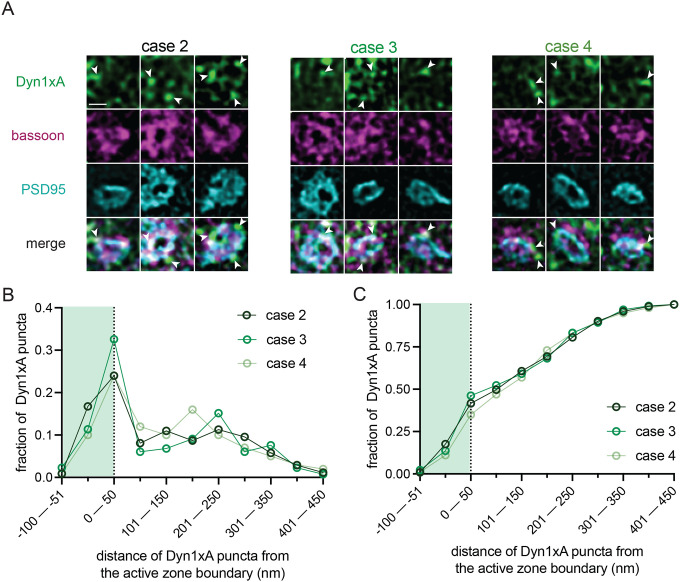
Dyn1xA clusters in human excitatory synapses. (A) Example STED images of endogenous Dyn1xA localizations (white arrowheads) in acute brain slices from three different humans. Scale bar: 300 nm. More example STED images provided in Figure S7. (B) The distribution of Dyn1xA puncta relative to the active zone edge, defined by Bassoon, analyzed in top view synapse images. Shaded region indicates area inside the active zone. See Data Table S1 for specific n values. (C) Cumulative plots of data presented in (B).

Tissue morphology was also largely preserved with the use of 15% PVP, and pearled axon morphologies were observed in human neocortical slices (Figure 4A). As with the mice, we stimulated human slices from two cases (1 female and 1 male) and froze them at 100 ms, 1 s, and 10 s. Unstimulated slices were used as controls. In both cases, uncoated pits appeared predominantly within 5–7 nm from the active zone, 100 ms after a single stimulus (Figure 4B–D, Figure S6A,B for more micrographs; median pit distances: 5.775 and 6.499 nm for case 1 and 2 respectively). Simultaneously, the number of large vesicles increased, indicating potential internalization of endocytic vesicles. Interestingly, the number of putative endosomes also peaked at 100 ms. To further probe the ultrafast endocytic pit formation timepoint, we froze two additional cases (2 males) at 100 ms with the necessary unstimulated controls. Similarly, these two cases exhibited uncoated pits within 6–10 nm from the active zone (Figure 4D; Figure S6C,D; median pit distances: 6.600 and 10.76 nm for case 3 and 4 respectively), 100 ms after a single stimulus (Figure 4B,C). Clathrin-coated pits were occasionally present, but did not increase in numbers after stimulation at the timepoints examined (Figure 4C; see Figure S6E for micrographs of uncoated pits versus observed clathrin-coated pits from this dataset for comparison). No clear Ω-figures were observed in these human samples. These data suggest that synaptic vesicle endocytosis in human cortical synapses may be mediated by ultrafast endocytosis on a millisecond timescale. To further examine a possible pathway conservation, we localized Dyn1xA in acute human brain slices, prepared on the same day (see Methods). Notably, STED imaging across three cases (1 female and 2 males; note that we could not recover enough tissue in one case) showed Dyn1xA puncta in PSD95-positive excitatory cortical synapses located predominantly at the active zone boundary and periactive zone (−50 nm to +50 nm) (Figure 5A,B; Figure S7 for example human slice STED overview and additional side view synapses that were present but not analyzed). Up to 46% of Dyn1xA signals localized within this region (Figure 5C; ~42% for case 2, ~46% for case 3, and ~35% for case 4). Altogether these data show that zap-and-freeze can be used to investigate the membrane dynamics of human brain tissues at millisecond and nanometer resolutions, and suggest that ultrafast endocytosis may be conserved in the human brain.

## Discussion

Here we present an approach to study membrane dynamics in human brain tissue. The zap board for a high-pressure freezer induces neuronal activity in acute mouse cerebellar slices detectable with calcium imaging. Ultrastructural analysis of mouse and human cortical synapses showcase similar uncoated pits forming ≤10 nm from the active zone 100 ms post-stimulus. Localizations of Dyn1xA near the endocytic zone of mouse and human cortical synapses suggest that these uncoated pits likely represent ultrafast endocytosis intermediates.

### Strengths and shortcomings of the method

The presented method has both advantages and disadvantages. A major advantage is that no exogenous proteins like channelrhodopsin are necessary. Therefore, we can attempt to study synaptic transmission in a more ‘native’, *ex vivo* context and in organisms or tissues not easily genetically manipulated. Patient tissues also maintain cell diversity, interactions, and cytoarchitectures that stem cell *in vitro* systems cannot provide^38^ and thus, may reveal cell-autonomous mechanisms occurring in neurons and non-cell autonomous events involving surrounding glia^39^. Zap-and-freeze may prove useful in examining post-mortem or surgical resections from patients affected by disease (provided short post-mortem time intervals). Many neurological and neurodegenerative diseases present synaptic dysfunction^40^ and endolysosomal defects^41^. Visualizing synaptic dynamics in human tissue will likely create more directly translatable results towards possible treatment developments, and may allow for the validation of cell biology mechanisms found in animal and cell models.

It is worth noting that this approach has limitations. We tested neocortical slices from male (n=3) and female (n=1) epilepsy patients spanning in age from 20 to 35. The parameters we measured seemed unaffected by sex assigned at birth or possible past brain traumas. However, we cannot dismiss the fact that this is likely due to the limited number of independent specimens. We also do not have access to genetic or other private patient information, which may affect the interpretation of results. From a technological standpoint, our theoretical calculations suggest that it would take an additional 3–4 ms for the core of brain slices to reach 0 °C^18^, leaving an ambiguity in the timing of the actual freeze relative to stimulation. This issue would not affect the method’s ability to capture synaptic vesicle endocytosis but presents a possible challenge for investigating exocytosis mechanisms that occur more quickly. Our use of 2D imaging is also probably excluding subtle, interesting information about intra-tissue connections, though we expect this technique can be easily adapted for use with existing 3D EM imaging. Furthermore, unlike optogenetic stimulation, most axons parallel to the electric field are likely stimulated, making it more difficult to study circuit-specific questions. Nonetheless, the potential benefits of this technique outweigh these challenges.

### Synaptic vesicle endocytosis mechanism

Mechanisms of synaptic vesicle recycling have been debated over the last 50 years. In the 1970’s, Heuser and Reese proposed clathrin-mediated endocytosis as the major pathway for synaptic vesicle recycling after intense stimulation of frog neuromuscular junctions^42^. Around the same time, Ceccarelli and his colleagues proposed another mechanism^34^, named kiss-and-run^43^. Over the years, evidence for both mechanisms has accumulated but the debate has continued^44^. Since the early observation of fast compensatory endocytosis in bipolar cells of fish^45^, we have presented evidence over the last decade for clathrin-independent, ultrafast endocytosis being the predominant mechanism in both *Caenorhabditis elegans* neuromuscular junctions and mouse hippocampal synapses^12,27,28,30–32,37^. Similarly, several groups have implicated the existence of ultrafast mechanisms at play in mammalian central synapses^46–49^. Our data here add to this hypothesis. Though we used a single stimulus to probe synaptic vesicle endocytosis, this stimulus is likely relevant given that the firing rate of some human cortical neurons can be very low based on the cognitive task being performed (<1 Hz)^50^. More importantly, the molecular mechanism may be conserved in human cortical synapses. As in cultured mouse hippocampal neurons, the endocytic protein Dyn1xA seems pre-localized to the endocytic zone, likely accelerating endocytosis at human synapses. Though mechanistic investigations of lab-based organisms will undoubtedly contribute to our understanding of synaptic transmission, direct examination of human tissues continue to provide key information.

## STAR Methods

### Lead contact

Further information and requests for resources and reagents should be directed to and will be fulfilled by the lead contact, Shigeki Watanabe (shigeki.watanabe@jhmi.edu).

### Data and code availability

All original code and data are available from the lead contact upon request.

Any additional information required to reanalyze the data reported in this paper is available from the lead contact upon request. The macros and Matlab scripts for EM image analysis are available at https://github.com/shigekiwatanabe/SynapsEM. Additional procedures are described in Watanabe et al. (2020)^51^. STED image analysis codes are available at https://github.com/imotolab-neuroem/STED_image_analysis_package_public_v1.4/tree/main.

#### Mice

All procedures involving mice were approved by the Johns Hopkins Animal Care and Use Committee and the regional authority of the state of Saxony, Germany (T09/16), following the guidelines of the National Institutes of Health and the European Communities Council. Both males and females were used in this study. All animals were kept on a 12 hour light/dark cycle and provided access to unlimited food and water. Wild-type mice used for slice experiments were obtained from Taconic (B6NTAC), kept at Johns Hopkins, and from in-house breeding (C57BL/6N) from the animal core facility at Leipzig University. *Dnm1* KO mice^52^ used for Dyn1xA antibody validation were obtained from Dr. Pietro De Camilli.

#### Human tissue

Patient samples were collected under a protocol by the Institutional Review Board (IRB) at Johns Hopkins Hospital from neurosurgical resections performed for neurologic disease, generally intractable epilepsy. Because the tissue resections occurred regardless of any research use, the IRB determined that waiver of consent was appropriate. After gross assessment by a neuropathologist, a small portion of diagnostically unnecessary neocortical tissue was de-identified and placed in room temperature NMDG containing artificial cerebrospinal fluid (aCSF) while transported to the lab—recipe described below.

#### Acute brain slice preparation

Acute mouse cerebellar slices were prepared from 21- to 26-day-old C57BL/6N mice of either sex (n=10) as described previously^24^. In short, under deep isoflurane anesthesia mice were decapitated. The skull was immediately opened, and the cerebellar vermis rapidly removed and placed in ice-cold aCSF_2 (in mM: 125 NaCl, 2.5 KCl, 2 CaCl_2_, 1 MgCl_2_, 1.25 NaH_2_PO4, 26 NaHCO3, 20 glucose, bubbled with 95% O_2_ and 5% CO_2_, pH 7.4 at room temperature). Using a vibratome (Leica VT1200S), 100 μm horizontal slices were sectioned and incubated in aCSF_2 at 35°C for 40 min before they were stored at room temperature until usage.

Both mouse and human acute cortical slices were cut to 100 μm on a vibratome (Leica VT1200S) in room temperature NMDG aCSF (in mM: 92 NMDG, 2.5 KCl, 1.25 NaH_2_PO4, 30 NaHCO_3_, 20 HEPES, 25 glucose, 2 thiourea, 5 Na-ascorbate, 3 Na-pyruvate, 10 NAC, 0.5 CaCl_2_·2H_2_O, and 10 MgSO_4_·7H_2_O). Brain slices were then transferred into a custom-built recovery chamber filled with continuously carbogenated NMDG solution at 37 °C and allowed to undergo an initial 12 min recovery. After 12 min, slices were moved into a new recovery chamber filled with continuously carbogenated HEPES holding aCSF (in mM: 92 NaCl, 2.5 KCl, 1.25 NaH_2_PO_4_, 30 NaHCO_3_, 20 HEPES, 25 glucose, 2 thiourea, 5 Na-ascorbate, 3 Na-pyruvate, 2 CaCl_2_·2H_2_O, and 2 MgSO_4_·7H_2_O) and allowed to undergo a second recovery for at least 4 hr at 37 °C. Slices were recovered to ensure electrical viability and responsiveness to later electric field stimulation—also to repair cut site damage to maximize the amount of maintained tissue morphology while imaging. All aCSF solutions were titrated to 7.3–7.4 pH, left at room temperature to avoid potential tissue shrinkage-expansion cycles that can affect tissue ultrastructure, and bubbled with carbogen gas (95% O_2_, 5% CO_2_) before use. Note that we did not perform the Na+ spike-in procedure associated with the original NMDG recovery method^26^ to prevent possible tissue overexcitation.

#### 2-photon calcium imaging on the zap board

For calcium imaging, cerebellar slices were incubated for 20 min with 4 μmol Fura-2, AM (solved in Pluronic F-127 20 % solution in DMSO) in a submerged recording chamber filled with aCSF_2. To ensure sufficient incubation, circulation of carbogenated aCSF_2 was stopped and carbogen supply was provided above the fluid with a sinter filter and a cardboard box on top to create an oxygen-rich environment. After incubation, excess dye was washed out for another 20 min with carbogenated, circulating aCSF_2. Slices were then transferred with a spatula and paintbrush to the zap board and assembled in aCSF_2 between two sapphire discs, separated by a 100 μm Mylar spacer. A rubber O-ring was placed on top of the assembly to imitate high-pressure freezing experiments. Electrical TTL-driven inputs to the zap board were driven by an EPC-10 USB patch clamp amplifier and Patchmaster software (HEKA Elektronik), and signal outputs digitized. Voltage for triggering the LED in the optocoupler was set to 2.55 V for slice experiments. Calcium imaging was performed with a 2-photon laser scanning microscope (BX61WI microscope and Fluoview 10 M scanner, Olympus), using a 50 mm, f1.2 camera objective (Zuiko, Auto-S OM-system, Olympus) and a mode-locked Ti:sapphire laser (Mai Tai DeepSee, Spectra-Physics, set to a center wavelength of 800 nm), controlled by Olympus Fluoview ASW software (version 04.01). Line scans were performed to test stimulation paradigms, at room temperature. The calcium signal after stimulation was calculated over background (dF/F_0_). Minimum Fura-2, AM signal was revealed by exponential fits. Data was normalized to the minimum for comparison with other slices.

#### Zap-and-freeze experiments

For zap-and-freeze of acute mouse and human brain slices, tissue was always tested the same day as collection. Slices were transported from recovery chambers to the high-pressure freezer in a petri-dish filled with room temperature aCSF (in mM: 125 NaCl, 2.5 KCl, 1.25 NaH_2_PO_4_, 25 NaHCO_3_, 10 glucose, 2 CaCl_2_·2H_2_O, and 2 MgSO_4_·7H_2_O; 7.3–7.4 pH). Slices were then trimmed into smaller pieces ~6 mm by hand using a razor blade. After trimming, slices were placed into freezing medium containing room temperature aCSF, supplemented with NBQX (3 mM), Bicuculline (3 mM), and 15% polyvinylpyrrolidone (PVP) as a cryoprotectant—slices were kept in this solution for less than 2 min as the freezing apparatus was assembled. 15% PVP was chosen and assembled in a specimen sandwich based on a method previously published for optogenetic stimulation of mouse brain slices using the high-pressure freezer^13^. The table and sample chamber of the high-pressure freezer were kept at 37 °C to ensure the physiological temperature of slices were maintained during experiments^53^. Unstimulated controls for each experiment were always frozen on the same day and originated from the same mouse or patient. The device was set such that samples were frozen at 0.1, 1 or 10 sec after the stimulus initiation. Cortical columns were always visually aligned perpendicular to the direction of the electric field to maintain consistency across samples (this ensured cortical layers were in parallel with the electric field).

#### Freeze substitution

Frozen samples were transferred under liquid nitrogen to an automated freeze substitution unit held at −90 °C (EM AFS2, Leica Microsystems). Using chilled tweezers, samples were moved into acetone to help disassemble the freezing apparatus. Sapphire disks with brain slices were then quickly moved into sample carriers containing 1% glutaraldehyde (GA) and 0.1% tannic acid (TA) in anhydrous acetone. The freeze substitution program was as follows: −90 °C for at least 36 hrs with samples in 1% GA, 0.1% TA, samples were then washed five times with pre-chilled acetone (30 min each), after washing the fixative solution was replaced with pre-chilled 2% OsO_4_ in acetone and the program was allowed to continue; −90 °C for 11 hrs with samples in 2% OsO_4_, +5°C per hour to −20 °C, −20 °C for 12 hrs, +10 °C per hour to +4 °C, hold at +4 °C.

#### Sample preparation for electron microscopy

After freeze substitution, samples removed from the AFS were washed six times with acetone (10 min each) and incubated with increasing levels of plastic (100% epon-araldite diluted with acetone: 30% for 2 hrs, 70% for 3 hrs, and 90% overnight at +4 °C). After plastic infiltration, samples were embedded in 100% epon-araldite resin (Araldite 4.4 g, Eponate 12 Resin 6.2 g, Dodecenyl Succinic Anhydride (DDSA) 12.2 g, and Benzyldimethylamine (BDMA) 0.8 ml) and cured for 48 hrs in a 60 °C oven. Serial 40-nm sections were then cut using an ultramicrotome (EM UC7, Leica microsystems) and collected onto 0.7% pioloform-coated single-slot copper grids. Sections were stained with 2.5% uranyl acetate in a 50–50 methanol-water solution.

#### Transmission electron microscopy

Images of synapses were acquired on a Hitachi 7600 transmission electron microscope at 80 kV and 80,000x magnification. Samples were blinded and given random names before imaging. At least 98–100 images were obtained per experimental timepoint. Low magnification images of tissues were obtained at 30,000x (mouse) and 40,000x (human) magnification and stitched together using the TrakEM2 plugin for Fiji^54^, https://github.com/trakem2, with final contrast adjustments made in Adobe Photoshop (v21.2.1).

#### Electron micrograph image analysis

Electron micrographs were manually analyzed using the published SynapsEM protocol^51^. Images from one experimental replicate were pooled into a single folder, randomized, and blinded using Matlab scripts. Synapses not containing a prominent postsynaptic density or those with poor morphology were manually excluded from analysis after this blinded randomization. Using custom Fiji macros, membrane and organelle features were annotated and exported as text files. Those text files were again imported into Matlab where the number and locations of the annotated features were calculated. For pit distribution from the active zone, the distance from the nearest edge of pits to the active zone was calculated. To minimize bias and error all annotated, randomized images were thoroughly checked and edited by at least one other member of the lab. Representative electron micrographs were adjusted in brightness and contrast to different degrees, rotated and cropped in Adobe Photoshop (v21.2.1) or Illustrator (v24.2.3). All Fiji macros and Matlab scripts are publicly available at https://github.com/shigekiwatanabe/SynapsEM.

#### Antibody generation

To generate the Dyn1xA antibody, the C-terminal Dyn1xA-specific residues [846–864, Cys-RSGQASPSRPESPRPPFDL] were custom synthesized and used for rabbit immunization (Pacific Immunology). The antibody was affinity purified using the antigen peptides.

#### Immunohistochemistry

For immunohistochemistry, adult mice were anesthetized with isoflurane, transcardially perfused with 4% paraformaldehyde (PFA) and decapitated, with their whole brain dissected into PBS. Whole brains were embedded in O.C.T. compound (Tissue-Tek), frozen on dry ice, and sectioned in a coronal orientation at 40 μm on a cryostat (Leica CM 3050S). Human brain slices were collected from the tissue remaining after zap-and-freeze experiments were completed—slices were kept at 100 μm and fixed in 4% PFA. Staining was performed on free-floating sections according to the protocol described in Kruzich, E, et al^55^. Slices were permeabilized and blocked with 10% Normal Goat Serum (NGS) and 1% Triton X-100 in PBS for 3 hrs at room temperature. Primary antibodies diluted in 10% NGS and 0.025% Triton X-100 in PBS were added to slices for 48 hrs at +4 °C. Primary antibodies included: FluoTag-X2 anti-PSD95 (1:100), Bassoon (1:100), and Dynamin1xA (1:100). After removal of primary antibodies, slices were washed four times with 0.025% Triton X-100 in PBS, 15 min each wash. Secondary antibodies were diluted in PBS and incubated with slices for 48 hrs at +4 °C. Secondary antibodies included: goat anti-rabbit Alexa Fluor 594 (1:100) and STAR 460L goat anti-mouse IgG (1:100). Slices were washed four times with PBS, then washed once with Milli-Q water before being mounted onto plain microscope slides (Globe Scientific) using a paintbrush. Tissue was allowed to dry until transparent under foil. ProLong Diamond Antifade Mountant was added directly onto slices, with a coverslip placed on top. Samples were left to dry at least overnight before STED imaging.

#### Stimulated emission depletion (STED) imaging

2D STED images were acquired on an Aberrior FACILITY line microscope using a 60x oil objective lens (NA = 1.42). The excitation wavelengths were set as: 640 nm, 561 nm, and 485 nm for imaging Atto-643, Alexa-594, and STAR460L labeled target respectively. Imaging was performed at 20 nm pixel size, 0.61 AU pinhole, dwell time 5 μs. The STED beam was set at 775 nm with power of 10–15% used.

#### STED image deconvolution, segmentation and analysis

STED images were exported in .obf format using LiGHTBOX software (Abberior). The remaining image processing was performed using a custom made MATLAB code package (Imoto et al. 2024^29^). Images were extracted from .obf files and converted to .tif format as unsigned 16-bit integers. The extracted images were normalized based on the minimum and maximum intensity values within each image and then blurred with a Gaussian filter with 1.2 pixel radius to reduce the Poisson noise. Subsequently, the images were deconvoluted twice using the two-step blinded deconvolution method. The initial point spread function (PSF) input was measured from the unspecific antibody signals of STAR 460L, Alexa 594, or Atto 643 in the STED images. The second PSF (enhanced PSF) input was chosen as the returned PSF from the initial run of blind deconvolution^56^. The enhanced PSF was used to deconvolute the STED images to be analyzed. Each time 10 iterations were performed. Series of the deconvoluted STED images were loaded to the segmentation script utilizing MIJ: Running ImageJ and Fiji within Matlab (Sage 2017, MATLAB Central File Exchange). All presynaptic boutons in each deconvoluted images were selected within 45×45-pixel regions of interest (ROIs) based on the Bassoon and Dyn1xA signals. Top view and side view presynapses are sorted via script with supervision based on Bassoon shape. Distance distribution analysis was performed on top view images only, since Dyn1xA puncta can appear to be in the middle of synapses when seen in side view.

The boundary of active zone or Dyn1xA puncta was identified as the contour that represents half of the intensity of each local maxima in the Bassoon channel. The Dyn1xA puncta were picked by calculating pixels of local maxima. The distances between the Dyn1xA puncta and active zone boundary were automatically calculated correspondingly. For this distance measurement, first, MATLAB distance2curve function (John D’Errico 2024, MATLAB Central File Exchange) calculated the distance between the local maxima pixel and all the points on the contour of the active zone or Dyn1xA cluster boundary. Next, the minimum distance for each local maxima pixel was selected. Signals crossing the ROIs and the Bassoon signals outside of the stained neurons were excluded from the analysis. The MATLAB scripts are available from github (https://github.com/imotolab-neuroem/STED_image_analysis_package_public_v1.4) or by request.

#### Electrophysiological recordings, analysis and biocytin imaging

Human brain slices were transferred to a submerged recording chamber, perfused and maintained at 34°C in solution containing (in mM): 125 NaCl, 2.5 KCl, 1.25 NaH_2_PO4, 25 NaHCO_3_, 2 CaCl_2_, 2 MgCl_2_, 10 glucose, saturated with 95% O_2_, 5% CO_2_. Whole-cell recordings from human cortical pyramidal neurons were visualized under an upright Zeiss microscope with infrared optics. Recording pipettes had resistances of 3–5 MΩ when filled with an internal solution containing (in mM): 120 K-gluconate, 15 KCl, 10 HEPES, 2 MgCl_2_, 0.2 EGTA, 4 Na_2_ATP, 0.3 Na_3_GTP, and 14 Tris-phosphocreatine (pH 7.3). Whole-cell current-clamp recordings were made with MultiClamp 700B and 1440A digitizer (Molecular Devices). Data were acquired and analyzed using pClamp 10.4 (Molecular Devices). Series resistance was monitored throughout the recording and controlled below 20 MΩ. Data was discarded when the series resistance varied by ≥ 20%. Recorded neurons were filled with 0.2% (w/v) biocytin. Then, the slices containing biocytin-filled neurons cross-linked with 4% PFA and stained with Streptavidin Alexa Fluor 555 conjugate (1:1,000). Fluorescent images were obtained using a confocal microscope (Zeiss LSM 900).

#### Western blotting

Mixed neuron cultures from the hippocampus and cortex were prepared from E18 wild-type and Dnm1 knockout mice and harvested at DIV14 and DIV21. Astrocytes were prepared from the cortex of E18 wild-type mice and harvested after two weeks. Cells were homogenized in RIPA buffer (Cell Signaling Technology) supplemented with cOmplete Mini Protease Inhibitor (Roche), and centrifuged at 14,000 × g for 10 min at +4 °C. Extracts were separated by SDS–PAGE and transferred onto Immobilon-FL membranes (Millipore Sigma). The membranes were blocked with 3% Bovine Serum Albumin (BSA) in PBS containing 0.05% Tween 20 (PBST) for 1 h, incubated overnight at +4 °C with primary antibodies in 3% BSA in PBST, and then incubated with second antibodies for 1 h at room temperature in 3% BSA in PBST and washed with PBST. The primary antibodies used were Dynamin1xA (1:2,000) and β-actin (1:2,000; rabbit). The secondary antibody was IRDye 800CW Goat anti-Rabbit IgG (1:4,000). Immunocomplexes were detected using an Odyssey infrared imaging system (Li-COR).

For Western blot confirmation of Dyn1xA antibody, whole brain lysates from a 1-year-old female wild-type mouse and human not affected with epilepsy were tested (Human Brain Whole Tissue Lysate (Adult Whole Normal), Novus Biologicals). Samples were run at 80 V, 90 min and transferred at 100 V, 80 min. The membrane was blocked with 5% milk in 0.05% PBST for 30 min, incubated overnight at +4 °C with primary antibodies in 5% milk/PBST, and then incubated with second antibodies for 1 h at room temperature in 0.05% PBST before being washed with PBST. The primary antibodies used were Dynamin1xA (1:5,000) and β-actin (1:2,000; mouse). The secondary antibodies were IRDye 800CW Goat anti-Mouse IgG and IRDye 680RD Goat anti-Rabbit IgG (both 1:30,000). Immunocomplexes were detected using an Odyssey infrared imaging system (Li-COR). Precision Plus Protein Dual Color Standard was used as a molecular weight ladder.

## Resource availability

All the materials and protocols will be shared by the lead contact (Shigeki Watanabe) upon request. Raw images in the manuscript will be uploaded to figshare.com upon publication.

## Supplementary Material

Supplement 1 Figure S1. Electrical circuit of Leica’s zap board.
(A) Leica’s zap board. R1: charging resistor, C: capacitor, PD: photodiode, T1 & T2: transistors in Darlington configuration, R2: base-biasing resistor, Chamber: recess for assembly of sapphire disks and Mylar spacer to hold and stimulate the specimen (cf. Figure 1C), V+ and V-: supply voltage (9 V), S+ and S-: stimulus output.
(B) Corresponding equivalent electrical circuit to the zap board. For 2-photon imaging, the PD was removed and replaced by connectors for an external optocoupler (OC, see part C).
(C) Equivalent electrical circuits for charging the capacitors (top left, connected to V+ and V− of the zap board), for the external optocoupler (bottom left, built from a second zap board), and the stimulus read out (right, connected to S+ and S− of the zap board). The charging circuit was controlled by a digital signal (TTL level) to the ‘charge’ input, the optocoupler via a digital to analog signal to the ‘zap’ input and electrically driven by a 9 V battery. The ‘stimulus readout’ was connected to an analog-to-digital converter, with an optional and variable resistor (R_load, var_) in parallel. 10 kΩ was used for panel E-I since resistance of a slice (R_slice_) was 12–15 kΩ.
(D) Sequence of signals used to externally operate the zap board. The TTL signal (‘charge’) activates the relays and allows the 9 V battery to charge the capacitors; ‘Vcap’ shows the voltage of the capacitors; the ‘zap’ signal activates the LED, which activates the PD, Darlington transistors and stimulus.
(E) Normalized peak stimulus amplitude (1 ms) versus charging time for R_load_ of 10 kΩ (other R values gave similar results). The gray area denotes the standard charging time used in this figure and Figure 1. The fit was forced to go from 0 to 1.
(F) Normalized peak stimulus amplitude (1 ms stimulus duration) versus delay time for R_load_ of 10 kΩ data, whereas other R_load_ values resulted in similar values. Double-exponential fit was forced to go from 1 to 0. The fast decay time τ_fast_ is likely due to charging of the analog-to-digital (AD) input (220 pF, HEKA instruments) via the leak current of the PD and Darlington transistors.
(G) Voltage output curves for continuous zap commands. The light intensity shone on the PD was varied from 2.4–4 V supply voltage for the LED (in 100 mV and 250 mV steps up to 3 and 4 V, respectively). Voltage outputs for 3.75 and 4 V overlap, because the LED emission saturated. Failures at 2–2.4 V were omitted for clarity. Data shown for 10 kΩ R_load_. The uneven curve patterns likely result from the nonlinear behavior of the circuit consisting of LED, PD and Darlington transistors.
(H) For each strength of LED illumination (‘LED command voltage’), the decay time of a single exponential fit to the data shown in G is plotted (the fit was restricted from the peak to the time at which 2 V was reached, to exclude the nonlinearities expected to arise when voltage across the capacitors dropped below the working range of the Darlington transistors). Failures are plotted as open/grey symbols. The grey line shows the light-dependent resistance of the PD (R_PD_), calculated from t=R*C, with C = 50 μF and R represented by the resistances of the AD input (1 MΩ, HEKA instruments) in parallel with R2 (479 W) plus R_PD_ (in series).
(I) Plot of stimulus strength over a train of 10 stimuli given at 50 Hz (R_load_ = 10kΩ). Inset: zoom-in on the peaks of superimposed stimuli.

Supplement 2 **Figure S2.** Additional EM images for Figure 2.
(A-C) Electron micrographs of acute mouse brain slices that have undergone zap-and-freeze at the indicated time points.
(D) An example clathrin-coated pit is shown compared to uncoated pits from this dataset. Scale bar: 100 nm.

Supplement 3 **Figure S3.** Validation of Dynamin1xA antibody
(A) Western blot showing Dyn1xA antibody specificity. Equal amounts (20 μg) of protein from astrocytes and neuron cultures from wild-type and Dynamin 1 (*Dnm1*) knockout mice at DIV14 and DIV21 were analyzed.
(B) Immunofluorescence images showing specific detection in primary neurons using the Dyn1xA antibody. Neuron cultures from wild-type and *Dnm1* knockout mice were analyzed at DIV14.
(C) Western blot showing Dyn1xA antibody reactivity with whole brain lysates from wild-type mouse and human (note: samples were not loaded with a specific protein concentration). Mouse lysate is from a 1-year-old female wild-type mouse. Human lysate is ‘Human Brain Whole Tissue Lysate (Adult Whole Normal)’ from Novus Biologicals, and not from a tested epilepsy patient used in this study.
(D) Sequence of the Dyn1xA C-terminus in mice and humans. Sequence used to generate the antibody is highlighted in red.

Supplement 4 **Figure S4.** Additional STED images for Figure 3.
(A) Overview 2D, three-color STED image of a cortical region in an acute mouse brain slice. Example side view (i and ii) and top view (iii and iv) synapses are highlighted as panels. Scale bar: 300 nm unless noted.
(B) Example Dyn1xA puncta in side view excitatory synapse images.

Supplement 5 **Figure S5.** Electrophysiological validation of human cortical slice viability after NMDG recovery.
(A) Photos of samples from each case on a vibratome. Scale bars: 1 cm.
(B) Representative traces from case 1 cortex pyramidal neuron in response to a series of 400 ms current stepping from −200 to +300 pA with increments of 50 pA. Inset: representative trace in response to +300 pA injection.
(C) The average number of action potentials generated in response to depolarizing current pulses. n = 10 cells from 4 slices; case 1.
(D) Typical spike of case 1 human cortical pyramidal neuron obtained at the normal resting membrane potential (RMP).
(E) Representative traces from case 2 cortex pyramidal neuron in response to a series of 400 ms current stepping from −200 to +300 pA with increments of 50 pA. Inset: representative trace in response to +300 pA injection.
(F) The average number of action potentials generated in response to depolarizing current pulses. n = 6 cells from 2 slices; case 2.
(G) Typical spike of case 2 human cortical pyramidal neuron obtained at the normal RMP.
(H) Confocal images of pyramidal neurons filled by biocytin post-recording from case 1 and case 2. Scale bars: 300 μm.

Supplement 6 **Figure S6.** Additional EM images for Figure 4.
(A-D) Electron micrographs of acute human brain slices that have undergone zap-and-freeze at the indicated time points.
(E) Example clathrin-coated pits are shown compared to uncoated pits from this dataset. Scale bar: 100 nm.

Supplement 7 **Figure S7.** Additional STED images for Figure 5.
(A) Overview 2D, three-color STED image of an acute human neocortical slice (from case 2). Example side view (i and ii) and top view (iii and iv) synapses are highlighted as panels. Scale bar: 300 nm unless noted.
(B) Example Dyn1xA puncta in side view excitatory synapse images. Note: some Dyn1xA puncta appear to be in the middle of Bassoon signals when observed from side views, and this is why top view images were analyzed.

Supplement 8

Supplement 9

Supplement 10
