# Supplementary material for: Ultrastructural membrane dynamics of mouse and human cortical synapses
Source: bioRxiv. 2024 Dec 26:2024.12.26.630393. Preprint. [Version 1] doi: 10.1101/2024.12.26.630393 (PMC11703259; doi:10.1101/2024.12.26.630393)
Supplement: Supplementary file 2 [file media-10.pdf]

Table S3.

Sample name	Sex assigned at birth	Age range	Relevant info
Case 1	M	35	Intractable epilepsy, history of childhood meningitis
Case 2	F	20	Intractable epilepsy, idiopathic
Case 3	M	30-35	Intractable epilepsy, history of childhood meningitis
Case 4	M	35	Intractable epilepsy, history of motor vehicle accident
