# Supplementary material for: Ultrastructural membrane dynamics of mouse and human cortical synapses
Source: bioRxiv. 2024 Dec 26:2024.12.26.630393. Preprint. [Version 1] doi: 10.1101/2024.12.26.630393 (PMC11703259; doi:10.1101/2024.12.26.630393)

Figure S2. Eddings, et al.

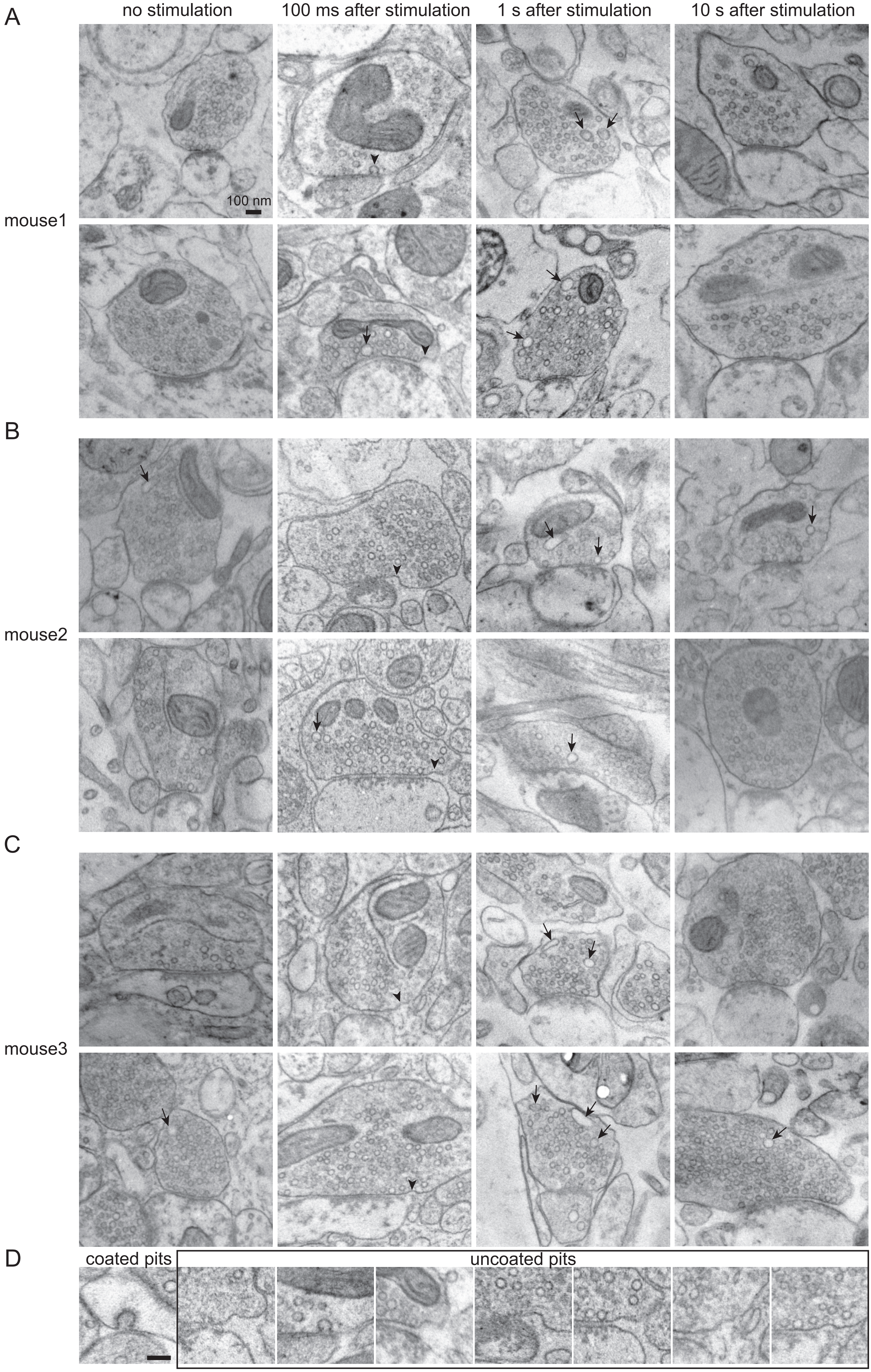

Supplement: Supplementary file 3 [file media-2.pdf]
