# Supplementary material for: Ultrastructural membrane dynamics of mouse and human cortical synapses
Source: bioRxiv. 2024 Dec 26:2024.12.26.630393. Preprint. [Version 1] doi: 10.1101/2024.12.26.630393 (PMC11703259; doi:10.1101/2024.12.26.630393)
Supplement: Supplementary file 4 [file media-3.pdf]

Figure S3. Eddings, et al.

A

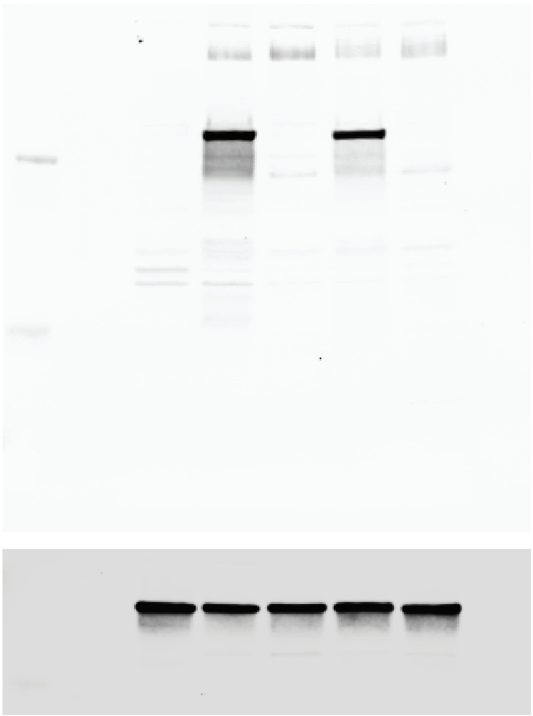

B

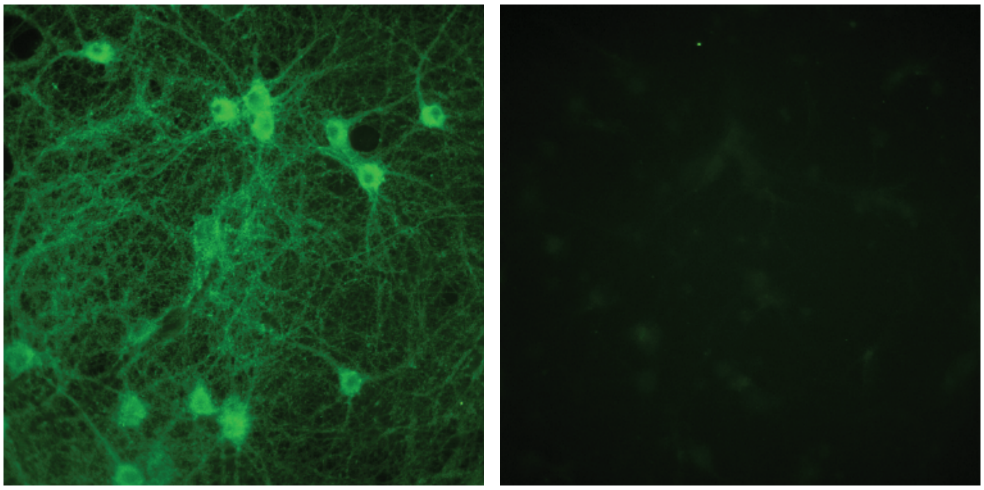

C

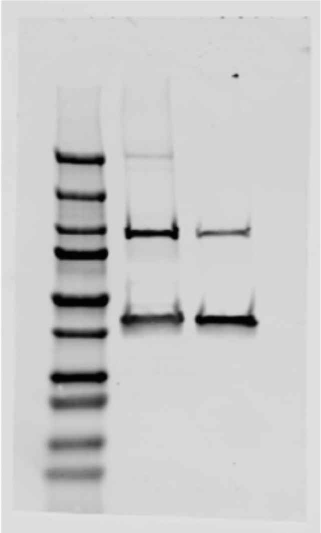

D

Homo

850 860
PGVPSRSGQA SPSRPESPRP PFDL

Mus

850 860
PGVPSRSGQA SPSRPESPRP PFDL
