# Supplementary material for: Ultrastructural membrane dynamics of mouse and human cortical synapses
Source: bioRxiv. 2024 Dec 26:2024.12.26.630393. Preprint. [Version 1] doi: 10.1101/2024.12.26.630393 (PMC11703259; doi:10.1101/2024.12.26.630393)
Supplement: Supplementary file 8 [file media-7.pdf]

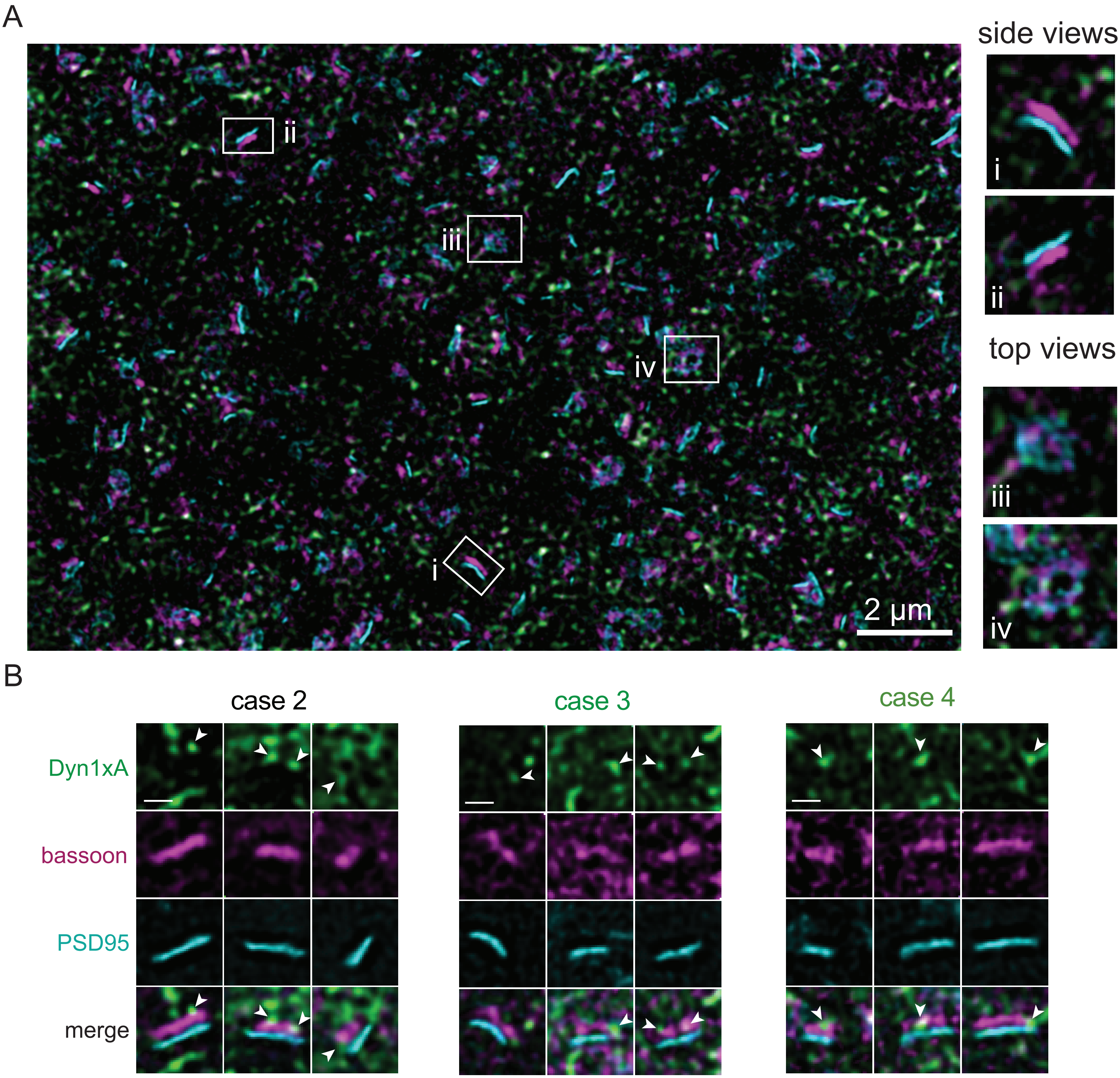
A
side views
ii
iii
iv
i
2 μm
i
ii
top views
iii
iv
B
case 2
case 3
case 4
Dyn1xA
bassoon
PSD95
merge
