# Supplementary material for: Ultrastructural membrane dynamics of mouse and human cortical synapses
Source: bioRxiv. 2024 Dec 26:2024.12.26.630393. Preprint. [Version 1] doi: 10.1101/2024.12.26.630393 (PMC11703259; doi:10.1101/2024.12.26.630393)
Supplement: Supplementary file 9 [file media-8.pdf]

Data Table S1.

Fig 1

Data of normalized Ca2+ signal vs. stimulus duration collected from n=10 slices from 2 independent animals.

Data of normalized Ca2+ signal vs. time after assembling the slice in the zap board chamber collected from n=11 slices from 2 independent animals.

Fig 2

All data are from 3 independent experiments from n = 3 acute mouse brain slices from 3 independent mice (2 female and 1 male) prepared and frozen on different days. Non-stimulated controls always frozen the same day.

Timepoint	No stim	100 ms	1 s	10 s
Total number of synaptic profiles analyzed	308	309	309	309

Fig 3

Region of interest top view images were obtained from n=3 slices from 3 independent mice (male and female).

Mouse	Number of top view synapses analyzed
1	440
2	424
3	404

Fig 4

All data are from 4 independent experiments from n = 4 acute human brain slices prepared and frozen on different days. Non-stimulated controls always frozen the same day.

Timepoint	No stim	100 ms	1 s	10 s
Number of synaptic profiles analyzed, Case 1	103	103	102	103
Number of synaptic profiles analyzed, Case 2	103	103	103	103
Number of synaptic profiles analyzed, Case 3	99	93	--	--
Number of synaptic profiles analyzed, Case 4	105	128	--	105

Fig 5

Region of interest top view images were obtained from n=1 slice each from 3 independent humans (1 female and 2 male).

**Case**	**Number of top view synapses analyzed**
2	346
3	132
4	100
