# Supplementary material for: Ultrastructural membrane dynamics of mouse and human cortical synapses
Source: bioRxiv. 2024 Dec 26:2024.12.26.630393. Preprint. [Version 1] doi: 10.1101/2024.12.26.630393 (PMC11703259; doi:10.1101/2024.12.26.630393)
Supplement: Supplementary file 10 [file media-9.pdf]

Table S2.

Electrophysiological properties of human cortical neurons from case 1

Number	RMP (mV)	Rm (MΩ)	Threshold (mV)	Amplitude (mV)
Cell 1	-60.00	64.70	-46.66	101.87
Cell 2	-60.00	95.80	-37.33	99.20
Cell 3	-65.00	103.67	-47.18	103.61
Cell 4	-59.00	89.10	-43.89	93.52
Cell 5	-71.13	91.55	-50.94	110.91
Cell 6	-72.67	137.95	-44.06	100.61
Cell 7	-65.00	66.50	-52.41	110.15
Cell 8	-62.00	122.10	-41.53	106.35
Cell 9	-68.00	117.80	-39.15	110.47
Cell 10	-65.67	118.40	-36.3	105.84
Mean ± SEM	-64.85±1.42	100.76±7.19	-43.93±1.73	104.25±1.69

Electrophysiological properties of human cortical neurons from case 2

Number	RMP (mV)	Rm (MΩ)	Threshold (mV)	Amplitude (mV)
Cell 1	-72.55	59.35	-52.44	106.39
Cell 2	-67.59	89.70	-48.24	113.80
Cell 3	-69.06	86.35	-49.73	114.00
Cell 4	-69.38	75.40	-58.43	106.89
Cell 5	-66.00	197.40	-48.16	117.10
Cell 6	-72.35	100.90	-51.74	119.67
Mean ± SEM	-69.49±0.97	101.52±18.28	-51.46±1.44	112.98±2.00

RMP: resting membrane potential
Rm: input resistant
